# Spatial distribution and relative biomass of bigheaded carps in Lake Balaton, Hungary estimated from an environmental DNA survey

**DOI:** 10.1371/journal.pone.0335950

**Published:** 2025-11-06

**Authors:** Nóra Boross, László Ardó, Duane C. Chapman, Gergely Boros, Zoltán Vitál, Viktor Tóth, Nathan L. Thompson, Katy E. Klymus, Catherine A. Richter

**Affiliations:** 1 HUN-REN Balaton Limnological Research Institute, Tihany, Hungary; 2 Hungarian University of Agricultural and Life Sciences, Department of Aquaculture and Environmental Safety, Research Center for Fisheries and Aquaculture, Szarvas, Hungary; 3 U.S. Geological Survey, Columbia Environmental Research Center, Columbia, Missouri, United States of America; Purdue University Fort Wayne, UNITED STATES OF AMERICA

## Abstract

Silver carp (*Hypophthalmichthys nobilis*), bighead carp (*H. molitrix*) and their hybrids, collectively known as bigheaded carps, have been introduced to Lake Balaton, Hungary. The current stock sizes are difficult to assess. We investigated environmental DNA (eDNA) techniques targeted for bigheaded carps, assessed the spatial distribution of eDNA in Lake Balaton, compared eDNA concentrations to environmental variables to assess potential habitat selection based on those variables, and provided an estimate of biomass of bigheaded carps relative to eDNA shedding rates per unit biomass observed in controlled experiments. Water samples were collected from 70 sites in an array across the lake. Biomass estimation was calculated using mean eDNA concentration obtained by quantitative PCR of the samples and previously determined eDNA shedding rates of bigheaded carps under controlled conditions in a laboratory. Concentration of eDNA was highly variable between sites, resulting in wide confidence intervals. Basins did not significantly differ in eDNA concentration, and there were no strong relationships between environmental variables and eDNA concentration, indications that bigheaded carps use the entire lake. The model provided an estimate of 4,830 metric tonnes (2,750–8,030 tonnes) of bigheaded carps in Lake Balaton, or 81.0 kg/ha. The eDNA method produced a value close to previous estimates by traditional means of total biomass of bigheaded carps in the lake, and like traditional methods, there was a broad confidence interval on the estimate of the mean. The results of the present study support the utility of aquatic eDNA analysis, and the need for further comparisons with fisheries methods and supporting data from laboratory studies.

## Introduction

Silver carp (*Hypophthalmichthys nobilis*) and bighead carp (*H. molitrix*), hereafter bigheaded carps, are planktivorous fishes native to the large rivers and lakes of Eastern Asia. Since 1950, they and their hybrids have been introduced into many countries across Asia, Europe and America [1,2], with the aim of increasing yields in aquaculture operations as well as in natural water fisheries, and to improve the water quality in eutrophic freshwaters [2,3]. Their establishment and spread outside their native range have caused various ecological problems, related mainly to alteration of the food web [4,5] and competition with native planktivore fishes [6–9].

Lake Balaton is an important recreational resource in Hungary, visited by millions of tourists every year. The lake provides vital and economically valuable ecosystem services such as recreational fishing, sailing, bathing, and drinking water for both residents and tourists. Over the past decades, Lake Balaton underwent significant changes in its trophic status [10]. During the period of 1960s to the 1990s, Lake Balaton experienced severe eutrophication, after which, as a result of the decline in external nutrient loadings, the lake has been returning to its former meso-eutrophic state [11].

Bigheaded carps were imported to Hungary for the first time in the 1960s from China and the former Soviet Union, and today they are ubiquitous in the country’s medium and large rivers [12]. In 1972, they were introduced into Lake Balaton to enhance fisheries and reduce serious planktonic eutrophication. However, water quality of the lake did not improve as a result of stockings of bigheaded carps and serious ecological problems occurred, as bigheaded carps consumed a significant quantity of zooplankton, which led to food competition with fry of native fish species [13,14]. Even though stocking was stopped by 1983, biomass of bigheaded carps seems to be still high in the lake, due to the low intensity of fishing, growth of individual fish, and recruitment from external sources [15]. Bigheaded carps in Lake Balaton are mostly hybrids of the two species [16] and therefore, the two species and their hybrids are hereafter referred to as a single “stock”. Morphological, histological and population genetic studies proved that the bigheaded carps in Lake Balaton do not regularly reproduce. Instead, abundance and biomass are sustained mainly by individuals escaped from surrounding ponds and aquaculture operations, and only occasional recruitment resulting from natural spawns is plausible [16,17]. The current stock is dominated by large, very robust, individuals [17].

Despite relatively detailed knowledge of the life history of bigheaded carps, only limited information is available about their biomass and spatial distribution in Lake Balaton. Commercial fishing has been prohibited in Lake Balaton since 2013, therefore there are no commercial catch reports to access for stock assessment. What can be known with confidence from the previous stocking reports is that approximately 290–350 tonnes of fingerlings were placed into the lake between 1972–1983 [18–20]. Estimations on the biomass of bigheaded carps has varied over a wide range, most probably due to the methodological difficulties in assessing accurate biomasses of evasive fishes in such a large and shallow lake. Bigheaded carps are notorious for their ability to avoid nets and electrofishing gear and for their reaction to moving boats [21–23]. Therefore, for the first time at Lake Balaton the present study used environmental DNA (eDNA) analysis to estimate the biomass and spatial distribution of bigheaded carps.

Presently, quantitative analysis of eDNA is being tested for estimating biomass and spatial distribution of fish (reviewed by Rourke et al. [24]). Analysis of eDNA involves extracting genetic material from the environment, e.g., from water, soil or sediment [25], not directly from the organisms. In the aquatic environment, eDNA can originate from the sloughing of skin cells, excretion/egestion, released gametes or from decomposing (decayed) individuals [25]. The DNA from these sources can be extracted from water samples and detected by polymerase chain reaction (PCR) [25]. Application of real-time quantitative PCR (qPCR) enables not only the detection of a species, but the estimation of the amount of eDNA in the original sample. This information can potentially allow one to infer biomass and spatial distribution of an organism as well [24]. eDNA concentration in water is determined by shedding and decay rates, which depend on several factors [26]. Shedding rate depends on the species, size and number of organisms (biomass), temperature, feeding, stress and physiological state of fish; whereas decay of eDNA is influenced by abiotic (sunlight, temperature, pH, salinity, flow rate) and biotic factors (extracellular enzymes and microorganisms) [26–30]. A number of models have been developed in order to estimate the biomass of aquatic organisms from eDNA quantity, with shedding and decay rates taken into consideration (e.g., [26,31,32]). Analysis of eDNA is relatively quick, non-invasive and does not require capturing individuals [25,33]. In some situations, it has been successfully applied to estimate biomass and spatial distribution of various freshwater and marine fish species (reviewed by Rourke et al. [24]).

The aim of the present study was to investigate eDNA analysis in order to characterize the stock of bigheaded carps in Lake Balaton. The spatial distribution of eDNA from bigheaded carps was assessed across the lake basins. Environmental variables, such as water temperature, chlorophyll-a (chl-a), Secchi depth, total suspended materials (TSM), water depth, and total dissolved solids (TDS), and their relationships with variation in eDNA concentrations across sampling sites were investigated to inform the understanding of habitat preferences of bigheaded carps in Lake Balaton. The hypothesis that the filter-feeding bigheaded carps would be more abundant, and thus shed more eDNA, in the more eutrophic basins of the lake was tested. A range of environmental variables was explored to ascertain any correlation with distribution of eDNA from bigheaded carps. For example, given the lake’s shallowness, the hypothesis that bigheaded carps would prefer areas with deeper water was tested. Additionally, an estimate of the total biomass of bigheaded carps in Lake Balaton was developed by quantification of eDNA from water samples in the present study relative to previous laboratory experiments with known biomass. This total biomass estimate is referred to as “relative biomass” to emphasize that it is based on a comparison with laboratory data and is not a direct measure of absolute biomass.

## Materials and methods

### Study area

**Fig 1:**
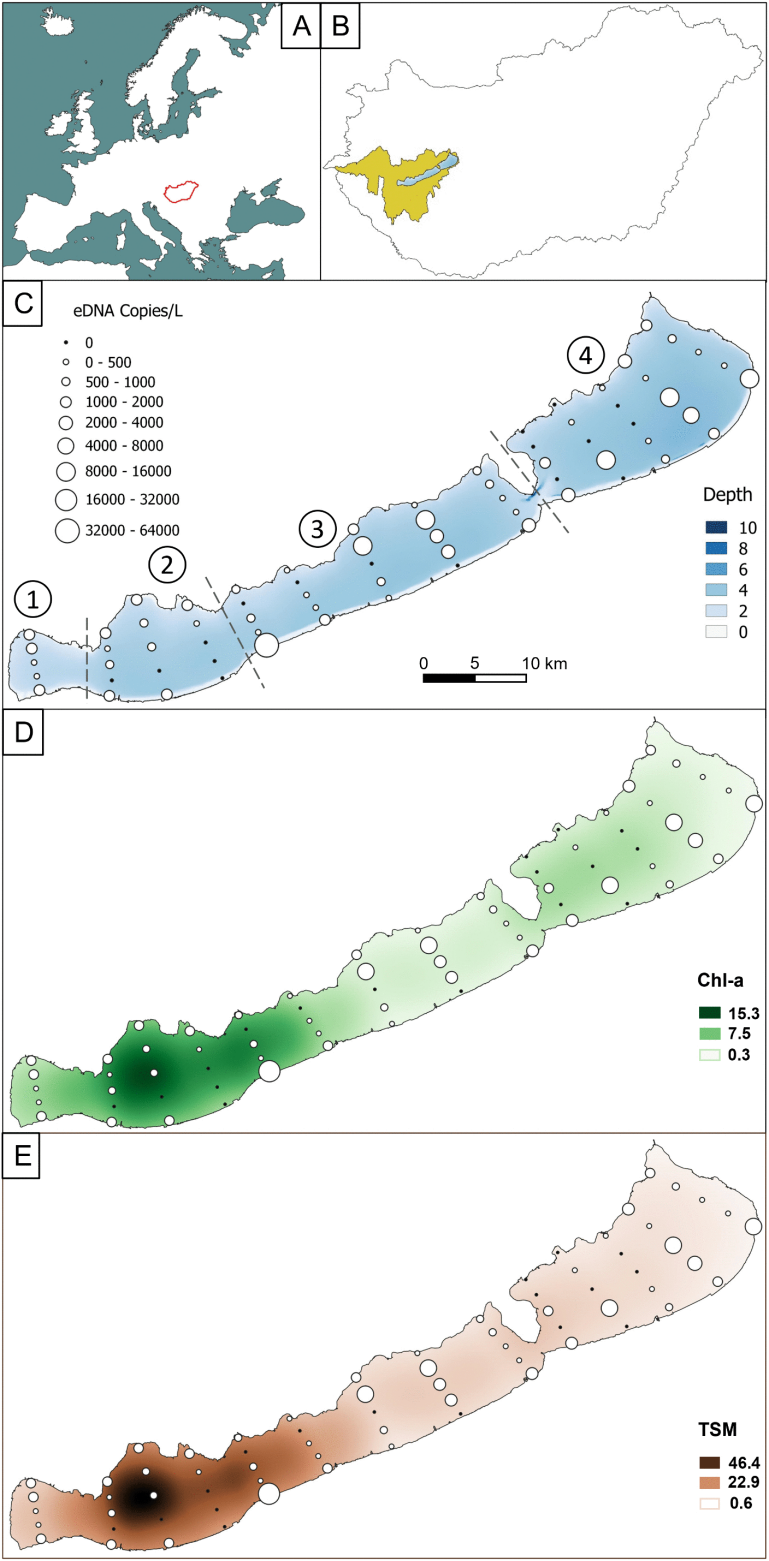
Environmental DNA concentrations (copies/L) from bigheaded carps in Lake Balaton, Hungary. The map of **(A)** Europe with Hungary outlined in red shows **(B)** Lake Balaton in blue with its watershed in yellow and (C) water depth (Depth **(m)**), (D) chlorophyll-a (Chl-a (µg/L)), and (E) total suspended material (TSM (mg/L)) indicated by color. Sampling sites are presented by circles, and eDNA concentration at each sampling site is indicated by the size of the circle. The four basins of the lake are labeled in panel C by numbers as follows: 1) Keszthelyi, 2) Szigligeti, 3) Szemesi and 4) Siófoki.

Lake Balaton is the largest lake in Central Europe, with a surface area of 596 km^2^ and a catchment area of 5775 km^2^ (Fig 1A and 1B). It is a shallow (mean depth ~3.3 m), mesotrophic lake, with strong wind-driven sediment resuspension, resulting in Secchi depth usually varying between 0.2 and 0.8 m [34]. Lake Balaton is characterized by four basins, from southwest to northeast: Keszthelyi, Szigligeti, Szemesi, and Siófoki. The basins exist in a gradient from eutrophic to mesotrophic from southwest to northeast, and are well connected except for a partial separation by the Tihany peninsula between the Szemesi and Siófoki basins.

The date for water sample collection – from 7:30 am to 5:30 pm, local time, on May 28^th^, 2018 – was chosen after 4 days of calm weather in order to minimise suspended sediments. Hence, there was clear and still water on the day of sample collection with an average Secchi depth of 1.38 m. Average water temperature was 24.5 °C. The HUN-REN Balaton Limnological Institute had been granted a permit for sampling in still and flowing water systems of Hungary for scientific purposes (permit reg. no.: PE-KTF/882-2/2018, valid between 25 January 2018 and 31 December 2027, issued by the Department of Environmental and Nature Conservation, Governmental Office of Pest County, Hungary).

### Inclusivity in global research

Additional information regarding the ethical, cultural, and scientific considerations specific to inclusivity in global research is included in the Supporting Information (S1 Checklist).

### Sample collection

Collection of small (50 ml) water samples that were centrifuged to concentrate particles bearing eDNA allowed rapid sample collection, avoid loss of sample due to clogging filters, for ease of DNA extraction, and because this method has been shown to be effective for bigheaded carp eDNA [27]. However, this method may have trade-offs in increased variation among water samples and lower total amounts of eDNA per sample compared to filtering larger volumes of water per sample. Water samples were collected from 70 sampling sites, situated along 12 transects in the lake (Fig 1C and S1 Table). Because the sites along transects were located at least a kilometer apart from each other, sampling sites were treated as independent. At each sampling site four replicate water samples were taken from about 10 cm below the water surface into separate 50 ml sterile conical centrifuge tubes, using a self-made sterilised devise to hold the tubes (S1 Fig). After sample collection, the pre-labelled tubes were immediately placed into a cooler box with ice water. Within 6 hours of collection, samples were placed into −20 °C freezers. Between each sampling site, the person handling the tubes changed sterilised gloves, and the sampling device and the cooler box were cleaned with 10% bleach, to avoid contamination in the field. Fifteen control field-blank samples were taken randomly along the sampling sites, when distilled water was poured into one of the pre-labelled conical tubes. Subsequently field-blanks were treated the same way as the rest of the samples. The meaning of sample IDs was withheld from laboratory personnel until after the samples were processed.

### Assessment of abiotic environmental parameters

During sample collection, water temperature and TDS were measured using an EXO-2^R^ sonde (YSI Incorporated), and Secchi-depths were also measured at each site with a Secchi-disk. Concentrations of chl-a (µg/L) and TSM (mg/L) in the water were estimated using Sentinel satellite images from May 26^th^, two days prior to sampling day, when images were clear and without any cloud coverage above the lake. The Sentinel-3 OLCI Level 1 images were downloaded from Copernicus Online Data Access (https://dataspace.copernicus.eu/browser/). After pre-processing and application of water pixel identification (Normalized Difference Water Index (NDWI), threshold = 0.6) [35], water chl-a and TSM data were retrieved from the images using the Case-2 Regional CoastColour (C2RCC) processor [36]. Actual water depths were calculated using the water level (data from the General Directorate of Water Management, https://www.ovf.hu/en/) according to a bathymetric model of Zlinszky et al. [37] for each sampling location using QGIS v.3.1, Volume Calculation Tool (REDcatch GmbH.) module.

### Extraction of DNA from water samples

Concentration of eDNA-bearing particles by centrifugation, and extraction of eDNA were as described [27]. Briefly, water samples were thawed at room temperature, then centrifuged at 3,700 x *g* at 4 °C for 30 min. Supernatants were removed and the settled pellets were air-dried for 20 min. DNA was extracted from the pellets using an E.Z.N.A. DNA Tissue Kit (Omega Bio-Tek), according to the manufacturer’s instructions. Each DNA sample was resuspended in 50 µl elution buffer. Prior to extraction, the working area was decontaminated with bleach, rinsed, and sterilized with 70 v/v % ethanol.

### Real-time quantitative PCR (qPCR) analysis

Each sample of DNA extracted from each water sample replicate was analysed in triplicate using real-time quantitative PCR (qPCR) on a LightCycler 96 (Roche) in 96-well white LightCycler 480 plates. Target sequences in eDNA samples were detected by ACTM1 primers and probe, which can detect both silver and/or bighead carp eDNA in one qPCR reaction, because the primer/probe set is complementary to a conserved region of the mitochondrial genome which is present in both silver carp and bighead carp but not in other fish [38]. A short, synthetic DNA sequence cloned into pIDTSMART vector (Integrated DNA Technologies) was added to each reaction at a constant concentration of 4315 copies/reaction as an internal positive control (IPC) to detect inhibition and false negatives. The ACTM1 probe was labelled with 6-FAM, whereas the IPC probe was labelled with HEX. Sequences of primers, probes, IPC and standard are presented in Table 1. In addition to the samples, a negative control (nuclease-free water), a positive control (genomic DNA extracted from silver carp fin) and six standards were run on each plate in triplicates. The standard was a 511 bp synthetic DNA fragment cloned into pIDTBlue vector (Integrated DNA Technologies), containing the target sequences for the ACTM1 primer/probe set. Tenfold dilutions were used for setting the standard curve with 10^6^ copies/µl as the highest concentration and 10^1^ copies/µl as the lowest one. Each reaction contained 500 nM of the ACTM1 forward and reverse primers, 125 nM of the ACTM1 probe, 125 nM of the IPC forward and reverse primers, and 93.75 nM of the IPC probe, 4315 copies IPC target, and 1X FastStart Essential DNA Probes master mix (Roche). Volumes of samples, standards, positive and negative controls were 2.5 µl in each reaction and the total reaction volume was 20 µl. Temperature cycling conditions were set as follows: 10 min preincubation at 95 °C followed by 40 cycles of 10 s at 95 °C and 30 s at 58 °C before holding at 4 °C. Analysis of run data was performed using LightCycler 96 software (Roche). A sample was considered positive if at least one of the three replicates was positive (sigmoidal amplification above the threshold within 40 cycles), and a run was considered valid if all three replicates of the negative control were negative. PCR efficiencies (E = 10^(−1/slope)) of the calibration curves varied between 1.91 and 2.00, and the R^2^ values were between 0.99 and 1.00. Starting quantities of each technical replicate in copies/reaction were calculated from the standard curve. Non-detects were included in the data set as zeros. Concentrations of eDNA were calculated based on the equation C = (SQ/D * E)/V, where C = the calculated eDNA concentration (copies/L), SQ = the starting quantity (copies/reaction), D = the sample volume per reaction (µl/reaction), E = the total extracted eDNA sample volume (µl), and V = the volume of water sampled (L). Concentrations for each water sample replicate were calculated as the mean of the three technical qPCR replicates (including zeros), and concentrations for each sampling site were calculated as the mean of the four water sample replicates per site (including zeros). The limit of detection was defined as the lowest concentration that could be detected in 95% of replicates, and was measured at 220 copies/L. Values below the limit of detection were reported as measured, and non-detections (zeros across all replicates) were reported as between 0 and 220 copies/L.

**Table 1:** Sequences of primers, probes, and standards for qPCR.

Name	Sequence
**ACTM1 forward**	5’ – GGC CGG AAC AGG ATG AAC AGT T – 3’
**ACTM1 reverse**	5’ – TAA TAG TTG TGG TGA TGA AGT TAA TTG – 3’
**ACTM1 probe**	5’ -/6-FAM/ CAC GCA GGA/ZEN/ GCA TCC GTA GAC CT/IABkFQ/ - 3’
**IPC**^**a**^ **forward**	5’ – TCT GAG TGT CCC TCG AAT CT – 3’
**IPC reverse**	5’ – GCA GTC CTT GAG AAC ATA GAG C – 3’
**IPC probe**	5’ -/HEX/ TGA CAG TCT/ZEN/ CCT TTC GTG TGA ACA TTC G/IABkFQ/ - 3’
**IPC target**	5’ – CTA CAT AAG TAA CAC CTT CTC ATG TCC AAA GCT CTC TGA GTG TCC CTC GAA TCT CAG ACG CTG TAT GAC AGT CTC CTT TCG TGT GAA CAT TCG GCT GCT CTA TGT TCT CAA GGA CTG CAC – 3’
**Standard**	5’ – GAG CTC AAA AGC TTT TGA CTC CTG CCC CCC TCT TTC CTT CTA CTA CTA GCC TCT TCT GGT GCT GAG GCC GGG GCC GGA ACA GGA TGA ACA GTT TAC CCG CCA CTC GCG GGT AAT CTT GCT CAC GCA GGA GCA TCC GTA GAC CTA ACA ATT TTC TCC CTC CAC TTA GCA GGT GTA TCA TCA ATT TTA GGG GCA ATT AAC TTC ATC ACC ACA ACT ATT AAC ATA GCT TTC GTT CAT TGA TTC CCC CTA TTT ACA GGA TAG AAT TCT ACT TTA AAC GAC ACC TGA ACA AAA ATC CAC TTC GGG GTA ATA TTC ATC GGC GTA AAT CTT ACA TTC TTC CCA CAA CAC TTC CTA GGT CTA GCA GGA ATG CCA CGA CGA TAC TCT GAC TAC CCA GAT GCC TAC GCC CTG TGA AAT ACA GTA TCA TCT ATC GGA TCT CTT ATT TCC CTG GTA GCA GTA ATT ATG TTC CTA TTT ATC CTA TGA GAA GCC TTC GCC GCT AAA CGA GGG ATC CAA GTC TAG A – 3’

^a^IPC, Internal Positive Control.

### Data analysis

All statistical procedures were conducted with the NADA2 package in R version 4.4.0 [39,40]. Mean, median, and standard deviation (sd) of total eDNA concentrations were calculated with the cenmle function. Outlier boundaries were ±3 sd from the mean. Upper and lower confidence limits (UCL and LCL) on the mean were estimated with the elnormAltCensored function. The resulting confidence interval describes the range that is predicted to contain the true mean in 95% of trials, given the observed data. Mean concentrations of eDNA from bigheaded carps were compared among basins with the cenanova procedure for an MLE test of mean natural logs [40]. Correlations of environmental variables with eDNA concentrations were tested by multiple regression analysis using the cencorreg procedure. Variables tested were water temperature, Secchi depth, water depth, TDS, TSM, and chl-a. Collinearity among explanatory variables was assessed using the vif function. The effects of log and cube root transformations of explanatory variables on the fit was explored with the partplots function. All possible models including a null model were tested, among all combinations of variables that were not co-linear. Normality of model residuals was tested for each model with the Shapiro-Francia test [41]. Explanatory power of models were compared with the AICctab function.

The total estimated relative biomass of bigheaded carps in Lake Balaton was calculated by dividing the total calculated eDNA shedding rate for Lake Balaton by the shedding rate per kg derived from a previous laboratory study [27,42]. The total shedding rate of eDNA from bigheaded carps was calculated based on an equation developed by [31]: S = (k + R/V) * C * V, where S is the calculated eDNA shedding rate (copies/day), k is a previously reported eDNA decay rate under basal conditions (0.877/day) [28], R is the flow rate of water (L/day), V is the total volume of water (L), and C is the measured mean eDNA concentration (copies/L). The same calculation was applied to a previously reported shedding rate for laboratory experiments using a known biomass of bigheaded carps under controlled conditions [27,42]. The mean eDNA shedding rate reported by [42] of 3,900 copies/kg/s had been corrected for flow rate but not degradation rate. The reported value was converted to copies/kg/day, then multiplied by the reported flow rate for the lab study of 456 L/day [27] to estimate a mean predicted eDNA concentration (C) for a tank containing 1 kg of bigheaded carps at 7.39 x 10^5^ copies/L. A shedding rate corrected for both water flow and eDNA degradation was then calculated using the previously described equation S = (k + R/V) * C * V [31], where k was the previously reported eDNA decay rate under basal conditions of 0.877/day [28], R was the reported flow rate of 456 L/day, and V was the reported water volume of 379 L [27]. The resulting shedding rate for bigheaded carps in the previous laboratory experiment was 5.83 x 10^8^ copies/kg/day.

## Results

### Spatial distribution of eDNA from bigheaded carps

**Fig 2:**
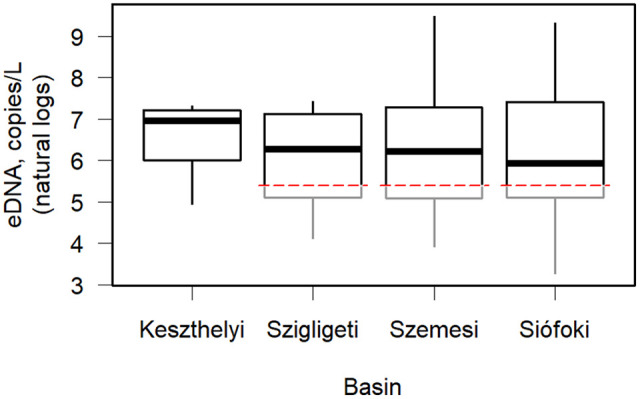
Environmental DNA (eDNA) concentrations of bigheaded carps, including silver carp (*Hypophthalmichthys nobilis*), bighead carp (*H. molitrix*), and their hybrids, in the four basins of Lake Balaton, Hungary. Data are presented as box plots on a natural log scale. Dashed red lines show the limit of detection (LOD) level of 220 copies/L, defined as the lowest concentration that can be detected with at least a 95% detection rate. Below the LOD, measured values were left unchanged and non-detects were imputed. All samples from the Keszthelyi basin had detectable eDNA.

Analyses of eDNA from bigheaded carps across the 70 sampling sites showed that their eDNA was distributed throughout Lake Balaton in a patchy distribution (Fig 1C). Some sampling locations had no detections, while others had high concentrations. No negative controls amplified and there was no evidence of inhibition. There was no significant difference in mean eDNA concentrations between the basins (Fig 2, Chi^2^ = 1.85, Df = 3, p = 0.61) but there was great variation between the sampling sites (mean = 2092.4 copies/L, median = 499.6 copies/L, sd = 8509.4 copies/L, LCL = 1042.4 copies/L, UCL = 3919.4 copies/L). Out of the 70 sampling sites 24% (17 sites) came back negative. One value failed the outlier test of ±3 standard deviations from the mean, a southern shoreline site in the Szemesi basin with 43,433 copies/L. This apparent outlier was removed. Summary statistics were recalculated without the outlier (mean = 1687.7 copies/L, median = 475.9 copies/L, sd = 5741.9 copies/L, LCL = 960.7 copies/L, UCL = 2806.9 copies/L). Subsequent analyses were conducted with the outlier removed from the dataset.

### Effects of environmental variables on concentrations of eDNA from bigheaded carps

Environmental variables were tested for possible effects on the observed spatial distribution pattern of eDNA from bigheaded carps. Water temperature, Secchi depth, water depth, TDS, TSM, and chl-a level were measured (Table 2, Fig 1D and 1E). The variables TSM and chl-a were co-linear, so they were not combined in the tested models. More complex multiple regression models did not improve the AIC scores substantially over the null model (Table 3).

**Table 2:** Summary of measurements of environmental variables water temperature (°C), Secchi depth (m), water depth (m), total dissolved solids (TDS, mg/L), total suspended materials (TSM, mg/L), and chlorophyll-a (chl-a, µg/L).

Variable	Mean	Standard Deviation	Minimum	Maximum	N
Chl-a (µg/L)	5.36	4.44	0.283	15.3	67
TSM (mg/L)	16.5	12.7	0.59	46.4	67
Water temperature (°C)	24.5	1.1	22.4	27.8	70
TDS (mg/L)	460	10.5	442	478	70
Secchi depth (m)	1.39	0.70	0.57	3.20	68
Water depth (m)	2.79	1.08	0.55	4.49	70

**Table 3:** Comparison of models of relationships of environmental variables with concentration of eDNA from bigheaded carps, including silver carp (*Hypophthalmichthys nobilis*), bighead carp (*H. molitrix*), and their hybrids (log copies/L).

Model	AICc^a^	dAICc^b^	df^c^	weight^d^
Total suspended materials (TSM, mg/L)	211	0	3	0.2403
Chlorophyll-a (Chl-a, µg/L)	212.4	1.4	3	0.1214
TSM+ total dissolved solids (TDS, mg/L)	212.7	1.7	4	0.1037
TSM+ Water depth (m)	213.2	2.2	4	0.0815
Water temperature (°C)	213.5	2.5	3	0.0702
Water depth	213.9	2.9	3	0.0574
Chl-a + TDS	214	3	4	0.0542
TDS	214.1	3.1	3	0.05
Secchi depth (m)	214.1	3.1	3	0.0499
Null	214.1	3.1	3	0.0499
Chl-a + Water depth	214.2	3.2	4	0.0482
TSM + TDS + Water depth	215	4	5	0.032
TDS + Water depth	216.1	5.1	4	0.0192
Chl-a + TDS + Water depth	216.2	5.2	5	0.0177
TSM + TDS + Secchi depth + Water depth + Water temperature	219.9	8.9	7	0.0028
Chl-a + TDS + Secchi depth + Water depth + Water temperature	221.1	10.1	7	0.0015

^a^Akaike information criterion corrected for small sample size (AICc).

^b^difference in AICc from the best model (dAICc).

^c^degrees of freedom (df).

^d^AICc weight, a measure of the relative likelihood of each model, range 0 to 1.0.

### Biomass estimation

An estimate of the total relative biomass of bigheaded carps in Lake Balaton was made by comparing the calculated mean eDNA shedding rate with a previously reported eDNA shedding rate from known biomasses of bigheaded carps. An overall eDNA shedding rate for bigheaded carps in Lake Balaton was calculated by applying the equation developed by [31]: S = (k + R/V) * C * V to the observed eDNA measurements. The outflow (R) from Lake Balaton of 5.4 x 10^8^ L/day [43] is negligible as a proportion of the total lake volume of 1.9 x 10^12^ L, so the equation simplified to S = k * C * V, where k was 0.877/day [28], V was the total volume of Lake Balaton, 1.9 x 10^12^ L, and C was the calculated eDNA concentration mean, LCL, or UCL. The estimated total shedding rate was mean 2.81 x 10^15^, LCL 1.60 x 10^15^, UCL 4.68 x 10^15^ copies/day. Dividing by the laboratory study shedding rate of 5.83 x 10^8^ copies/day/kg gave estimated relative total bigheaded carps biomass of mean 4.83 x 10^6^ kg, LCL 2.75 x 10^6^ kg, UCL 8.03 x 10^6^ kg. For comparisons with previous estimates, the estimate in kg was converted to metric tonnes by dividing by 1000: mean 4,830 tonnes, LCL 2,750 tonnes, UCL 8,030 tonnes. This calculation is an attempt to estimate an approximate level of the biomass that could provide a comparison for later studies and the results standing by themselves should be treated cautiously.

## Discussion

There was a high variance in eDNA copies between sites, which is similar to other studies [44] and is also observed in laboratory studies of eDNA [27]. High variance between sites seems to be common for eDNA studies and in this way eDNA studies are similar to traditional fishery methods. Bigheaded carps exhibit strong schooling behavior that is consistent with the observed pattern [2,45]. Still, besides the obvious reason for variance in eDNA copies detected (differences in local abundance of the target species), there are other factors, such as a clumpy distribution of eDNA-bearing particles, currents, wind, or non-live-fish sources of eDNA that might influence local eDNA concentration [42]. For example, in the case of the one extremely high outlier value, we suspect but cannot confirm that the sample was affected by a decaying carcass in proximity to the sample site. This sample site was in a very shallow and downwind location, which does not seem like a likely hotspot for large numbers of carp but does seem like a location to which a decaying carcass could have been transported by wind and current. The data analysis is presented excluding this outlier, with the note that including it did not substantially change the conclusions.

No substantial difference in eDNA concentration was observed between the basins of Lake Balaton. This was somewhat surprising because there is a trophic gradient along the longitudinal axis of the lake, with a decreasing level from the western to the eastern basin, as shown with long term data in [10]. It would seem logical that bigheaded carps would therefore select areas with higher productivity. There are many factors other than productivity that might influence large-scale carp habitat selection. Disturbance by recreational activity, preference for deeper water, competition with other bigheaded carps or with other fishes, or other unidentified factors could be responsible [45]. However, the results of the present study showed a lack of statistically significant differences among basins, and very small effects of environmental variables on eDNA concentrations. Thus, these results are not consistent with strong preferences by bigheaded carps for particular basins within Lake Balaton despite the gradient in limnological conditions across basins. Instead, the results of the present study suggest that the carp are utilizing the entire lake.

The measured environmental variables did not have substantial effects on bigheaded carp eDNA concentration. Bigheaded carps are highly mobile, do not seem to show a high loyalty to any home range and can move as local conditions change to follow food abundance [46–48]. However, because bigheaded carps are filter-feeders [2] the presence of suspended inorganic materials is likely to interfere with the filter-feeding process and result in the consumption of indigestible material. Lake Balaton is famous for high concentrations of suspended carbonate materials, which are resuspended from the sediments by wind and only slowly precipitate from the water column, resulting in a milky coloration of the lake, especially in windy periods [49]. These particulates are likely to interfere energetically and with digestion of foods consumed by filter-feeding, therefore it could be expected that high concentrations of particulates would be avoided by bigheaded carp. Mozsár et al. [14] noted a usually high ingestion of inorganic particulates in Lake Balaton bigheaded carp, an indication that bigheaded carps cannot avoid filtering the suspended carbonates in Lake Balaton and that it is likely an important physiological factor in the bigheaded carps of the lake. The lack of relationship between eDNA and particulate concentration suggests that wind-driven resuspension of eDNA from the sediment was not a substantial artifactual driver in this study. In addition, any relationships between wind-driven suspended sediments, fish habitat selection, and eDNA concentrations would have been minimized by the selection of a period of calm weather for eDNA sampling.

Estimation of relative fish biomass using eDNA is based on the shedding rate of detectable DNA into the environment [27], and the degradation rate [28] and fate of that DNA after it has been shed. The science of biomass estimation of aquatic species using eDNA is still developing, but early efforts have been encouraging [50]. Rourke et al. [24] found that 90% of 63 studies between 2012 and 2020 found a positive relationship between eDNA and fish biomass. Despite increasing research into the factors that control eDNA shedding and degradation, they are still inadequately understood, and this lack of knowledge limits confidence in biomass estimates. Shedding rates can be affected by many factors such as fish size, temperature, stress, or feeding status [26,27,29]. Degradation rates can vary with temperature, microbial activity, or water chemistry [28,29]. Many have espoused caution in use of eDNA methods to estimate biomass [51].

On the other hand, traditional fishery methods for stock assessments, and also newer methods such as hydroacoustics, are also affected by sometimes uncontrollable limitations on accuracy and precision. Traditional capture methods used in stock assessments are limited by habitat variables, and all gears are size-selective, such that biases in size composition are to be expected [52]. Traditional fisheries stock assessments generally ignore very small, young, fish that may often be a substantial part of the biomass by virtue of their high number, but are subject to high mortality such that their contribution to the biomass may change rapidly over a season. However, this factor is probably not substantial in Lake Balaton because natural reproduction of bigheaded carp, if it occurs at all, is likely a minor factor [16].

Capture-recapture studies are affected by tag retention and detection, mortality of tagged fish that differs from untagged fish, and differing behavior of tagged and untagged fish [53], and the extent that these factors affect stock assessments is rarely understood. Bigheaded carps are especially difficult to handle because of their tendency to injure themselves in nets or by their jumping behavior when held in tanks for implantation. Estimations of the stock of bigheaded carps in Lake Balaton showed that by the end of 2000s, a minimum of 1150 tonnes of bigheaded carps were present in the lake [19], while Tátrai et al. [18] assessed their biomass to be 4000–5000 tonnes during the same period, which could be equivalent to the one third of the entire fish biomass. The authors know of only one attempt at using capture-recapture methods for stock assessment of bigheaded carps [21], and no published studies of tag retention and survival of tagged wild-caught bigheaded carp. Implantation of telemetry receivers are more invasive than the tags used in capture – recapture studies, but low detection of telemetered bigheaded carps after early initial detections, or continuous detection in the same location (an indication of mortality) has affected many studies: [48,54–56]).

Hydroacoustics have been used with some success to estimate abundance and sizes of bigheaded carps [57]. However, Lake Balaton’s shallowness and the propensity of bigheaded carps to flee vessels [58] contraindicate use of hydroacoustics. Furthermore, the many large common carp (*Cyprinus carpio*) and wels catfish (*Silurus glanis*) which are present would be indistinguishable from bigheaded carps with most hydroacoustic methods.

Traditional fisheries stock assessments arrive at biomass estimates by estimating both the size and number of fishes, and calculating biomass from those values. In contrast, eDNA analysis provides only a relative biomass estimate by comparing eDNA concentrations observed in the field to 1) traditional assessments, 2) concentrations observed in laboratory or mesocosm experiments with known absolute biomass, or 3) modelled results based on eDNA shedding and decay rates measured under controlled conditions. eDNA studies can complement but not replace traditional assessments, because often it is necessary to know population numbers and size of fish present, and at least one of those values must be measured by a non-eDNA assessment.

Various traditional fisheries methods have been used to characterize the fish stock of Lake Balaton over time, and the results of these studies reflect the history of changes in the lake. Bíró [59] modelled the material flow of Lake Balaton from the perspective of fish, based on the data regarding material and energy flow along the food web. The model estimated an average fish biomass of 290 kg/ha for the lake by the end of the 1980s and early 1990s, representing a total fish stock size of 17,197 tonnes. Due to a significant decrease in the food base, there was a drastic reduction in total fish biomass at the end of the 1990s, which is also reflected in the following estimates.

Modern hydroacoustic fish stock assessments in Lake Balaton were first conducted by Kubecka et al. [60], who estimated the fish density in open water at an average of 169 ± 112 kg/ha. This same value was reported by Tátrai et al. [61] in their survey, which indicated a value of 170 ± 130 kg/ha in 2004. The significant margin of error in these estimates also indicates how patchy the distribution of fish stock is within the lake. Due to this patchy distribution more accurate estimates of stock size could only be achieved through surveys conducted with high regularity over large areas. This is supported by studies conducted between 2003 and 2007, where the average fish biomass values for open water varied significantly and were recorded as 34–114 kg/ha in the Siófok basin, 73–137 kg/ha in the Szemes basin, and 113–319 kg/ha in the Keszthely basin [62].

The eDNA shedding model provided an estimate of 4,830 metric tonnes (2,750–8,030 tonnes) of bigheaded carps in Lake Balaton, or 81.0 kg/ha. This value was in close agreement with previous traditional estimates [18] in the early 2000s, and the confidence intervals strongly overlapped. The confidence interval that the model provided was quite wide, due to the large variation in observed eDNA concentration across the sites. A value of 81.0 kg/ha is not an extraordinarily high value for bigheaded carp, which can reach extremely large biomass in natural systems [21]. Chapman [63] recorded a capture of 896 kg/ha of wild bigheaded carps from a shallow 122 ha lake in Missouri, USA, and that was not a complete harvest of all bigheaded carps in the lake. Lake Balaton is a mesotrophic lake [10] which should have the capacity to support a high biomass of bigheaded carp. As noted previously, both eDNA analysis and traditional fisheries assessments are subject to biases and high variability. The eDNA shedding model is an attempt to generate an approximation of the biomass that may exist in the lake as a comparison for later studies. These results should be treated cautiously. Further advances in the use of DNA to estimate biomass may result in improvements to the model that may result in more accuracy and precision of the method. In particular, the shedding rate and degradation rate used from previous laboratory studies had great influence on the resulting relative biomass estimate, and may not be accurate for conditions in Lake Balaton. Future studies that measure these parameters under controlled conditions directly relevant to Lake Balaton could improve current and future estimates of biomass. Challenges in measuring shedding rates include housing the large fish typical of the Lake Balaton stock, changes in fish behavior between wild and captive conditions, differences in diet in captivity, and stress from captivity altering eDNA shedding rates. Challenges in measuring degradation rates include finding a suitable source material that has a high DNA concentration but is representative of eDNA naturally shed from wild fish, and maintaining environmental conditions in the laboratory that are representative of Lake Balaton.

## Conclusion

Absolute biomass estimates of aquatic organisms in the field are often difficult or impossible. Relative biomass estimates are strongly influenced by the method used to generate them. We used a novel method, eDNA quantification, to generate a biomass estimate which was in the range of other biomass estimates from more traditional methods. eDNA was found throughout the lake and there were no strong and obvious relationships with basin or with environmental variables, an indication that bigheaded carps use the entire lake. The present study illustrates the potential for eDNA monitoring to provide relative biomass estimates over time, feedback on effects of management actions, and comparisons among different systems with populations of bigheaded carps. Further use of eDNA monitoring will help refine methods and interpretation of results.

## Supporting information

S1 Fig Device for water collection. (PNG)

S1 Table Coordinates of Sampling Locations at Lake Balaton and raw data including environmental variables water temperature (°C), Secchi depth (m), water depth (m), total dissolved solids (TDS, mg/L), total suspended materials (TSM, mg/L), and chlorophyll-a (chl-a, µg/L); and concentration of eDNA from bigheaded carps, including silver carp (*Hypophthalmichthys nobilis*), bighead carp (*H. molitrix*), and their hybrids (copies/L). (CSV)

S1 Checklist Inclusivity in global research questionnaire. (DOCX)
