# Supplementary figures and images for: Spatial distribution and relative biomass of bigheaded carps in Lake Balaton, Hungary estimated from an environmental DNA survey
Source: PLoS One. 2025 Nov 6;20(11):e0335950. doi: 10.1371/journal.pone.0335950 (PMC12591465; doi:10.1371/journal.pone.0335950)

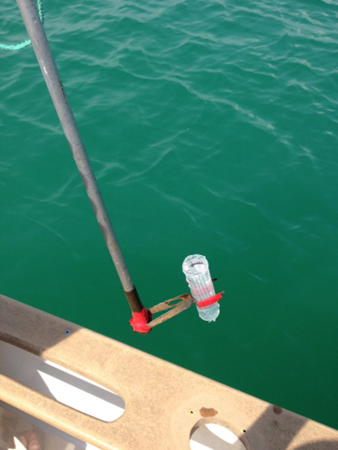

Supplement: S1 Fig — (PNG) [file pone.0335950.s001.png]
